# Prevention of respiratory syncytial virus disease across the lifespan in Italy

**DOI:** 10.1186/s41479-025-00160-4

**Published:** 2025-04-05

**Authors:** Paolo Manzoni, Eugenio Baraldi, Irene Cetin, Stefania Maggi, Matteo Riccò, Roberta Siliquini, Giovanni Sotgiu, Elsa Viora

**Affiliations:** 1 Department of Public Health and Pediatric Sciences, University of Torino School of Medicine, Torino, 10125 Italy; 2 Department of Maternal-Infant Medicine, “Degli Infermi” Hospital, University of Torino School of Medicine, via dei Ponderanesi, 2, Ponderano, Biella, BI 13875 Italy; 3 Neonatal Intensive Care Unit, Department of Woman’s and Child’s Health, University Hospital of Padova, Padova, Italy; 4 Institute of Pediatric Research “Città della Speranza”, Padova, Italy; 5 Department of Clinical and Community Sciences, Università degli Studi di Milano, Milano, 20157 Italy; 6 Fondazione IRCCS Ca’ Granda, Hospital Maggiore Policlinico, University of Milan, Milano, 20122 Italy; 7 Neuroscience Institute, Aging Branch, National Research Council, Padova, Italy; 8 AUSL-IRCCS di Reggio Emilia, Service for Health and Safety Prevention in the Workplaces (SPSAL), Local Health Unit of Reggio Emilia, Reggio Emilia, Italy; 9 Department of Public Health and Pediatric Sciences, University of Turin, Torino, 10126 Italy; 10 AOU Città Della Salute e Della Scienza, Torino, Italy; 11 Clinical Epidemiology and Medical Statistics Unit, Department of Medical, Surgical and Experimental Sciences, University of Sassari, Sassari, Italy; 12 (Retired) Obstetrics-Gynecological Ultrasound and Prenatal Diagnosis Unit, Department of Obstetrics and Gynecology, AOU Città Della Salute e Della Scienza, Torino, Italy

**Keywords:** Respiratory syncytial virus, Respiratory tract infection, Preventive measures, Passive immunization, Monoclonal antibody, Vaccine, Newborns and infants, Pregnancy, Older adults, High-risk populations

## Abstract

Respiratory syncytial virus (RSV) causes substantial morbidity and mortality across the lifespan, with the highest burden seen in infants and older adults. Recently approved immunizing agents, including long-acting neutralizing monoclonal antibodies and a maternal vaccine for passive immunization of newborns, and three vaccines for adults aged 60 years and older who are vulnerable to RSV disease, have the potential to prevent severe RSV-associated disease if implemented successfully. The use of these agents will be implemented in some Italian regions over the next few months, although no consistent timelines or decisions for adoption at the national level are expected. A multidisciplinary group of experts in neonatology, obstetrics and gynecology, respiratory medicine, geriatric medicine, hygiene, and public health reviewed the evidence on RSV prevention and present here their considerations on implementing an RSV prevention strategy in Italy. Given the associated disease burden, it is essential to move quickly to deploy these agents in vulnerable populations, enhance surveillance to accurately detect/predict seasonal trends in RSV activity and measure the impact of prevention strategies. Continuing research combined with widespread use of more sensitive testing is needed to identify vulnerable populations and risk factors. Policies are needed to support these preventive measures in the Italian healthcare system, and access must be accompanied by educational initiatives and advocacy to promote acceptance by HCPs and the target population.

## Introduction

Respiratory syncytial virus (RSV) is a highly contagious enveloped RNA virus belonging to the Pneumoviridae family [1], that is primarily transmitted through respiratory droplets or contact with secretions on contaminated surfaces. Immune responses to natural RSV infection provide only transient protection, and re-infections are frequent in children and adults [2, 3]. While RSV is a common cause of upper respiratory tract infection, the lower respiratory tract may also be involved [4–6]. Bronchiolitis or pneumonia requiring hospital admission, hydration, supplemental oxygen, and ventilatory support can be the outcome of early and severe RSV infection in infancy; these infants are also at increased risk of wheezing or asthma later in childhood [7–12]. Long-term consequences of lower respiratory tract infection (LRTI) in adults may include chronic obstructive pulmonary disease (COPD) exacerbations and accelerated deterioration of lung function [13], increased mortality in patients with congestive heart failure, with or without exacerbations [14], and deterioration of functional status [15]. In addition, RSV infection early in life can predispose to COPD [16].

Until recently, only one pharmacological preventive tool was available–the monoclonal antibody (mAb) palivizumab–but its high cost and need for monthly administration during the RSV season has limited its recommendation to children at high risk for severe disease (i.e., children born at ≤ 35 weeks of gestation who are less than 6 months old at the onset of the RSV season, those less than 2 years of age and requiring treatment for bronchopulmonary dysplasia within the last 6 months, or those less than 2 years of age with hemodynamically significant congenital heart disease). The recent approval of effective new immunizing agents, including a maternal vaccine for passive immunization of newborns following administration during pregnancy, an effective long-acting neutralizing mAb, and three vaccines for older adults, has the potential to make severe RSV-associated LRTI an immunization-preventable disease. Guidelines and policies are needed to support these preventive measures (Table 1). As of September 2024, the use of these agents to protect vulnerable populations in Italy has been introduced in several regions but has not been formally implemented at the national level.

**Table 1:** Agents approved in Europe for active or passive immunization against RSV

Agent	Indication	Schedule
**Maternal vaccine (passive immunization of infants)**		
Abrysvo™ [17] (RSVPreF) RSV vaccine (bivalent, recombinant) RSV subgroup A stabilized prefusion F antigen, and RSV subgroup B stabilized prefusion F antigen	Passive protection against lower respiratory tract disease caused by respiratory syncytial virus (RSV) in infants from birth through 6 months of age following maternal immunization during pregnancy.	A single dose should be administered to pregnant individuals between 24–36 weeks of gestation (or between 28–36 or 32–36 weeks, depending on the local official recommendations in each EU country)
**mAbs (passive immunization of infants)**		
Synagis™ [18] (palivizumab) recombinant humanized mAb	Prevention of serious lower respiratory tract disease requiring hospitalization caused by respiratory syncytial virus (RSV) in children at high risk for RSV disease: • Children born at 35 weeks of gestation or less and less than 6 months of age at the onset of the RSV season. • Children less than 2 years of age and requiring treatment for bronchopulmonary dysplasia within the last 6 months. • Children less than 2 years of age and with hemodynamically significant congenital heart disease.	Given once a month during seasonal RSV epidemic period
Beyfortus™ [19] (nirsevimab) human IgG1κ mAb	Prevention of Respiratory Syncytial Virus (RSV) lower respiratory tract disease in neonates and infants during their first RSV season.	Administer a single dose prior to start of the RSV season, or from birth for infants born during the season
**Immunization of older adults and high-risk groups**		
Abrysvo™ [17] (RSVPreF) RSV vaccine (bivalent, recombinant) RSV subgroup A stabilized prefusion F antigen, and RSV subgroup B stabilized prefusion F antigen	Active immunization of individuals 60 years of age and older for the prevention of lower respiratory tract disease caused by RSV.	A single dose should be administered
Arexvy™ [20] (RSVPreF3) RSV vaccine (recombinant, adjuvanted) RSV subgroup A stabilized prefusion F antigen	Active immunization for the prevention of lower respiratory tract disease (LRTD) caused by respiratory syncytial virus in adults 60 years of age and older.	Administer as a single dose
mResvia [21] (mRNA-1345)	Active immunization for the prevention of lower respiratory tract disease (LRTD) caused by Respiratory Syncytial Virus in adults 60 years of age and older.	Administer as a single dose

*EU* European Union, *IgG1κ* immunoglobulin G1 kappa, *LRTD* lower respiratory tract disease, *mAb* monoclonal antibody, *RSV* respiratory syncytial virus

A multidisciplinary group of experts comprising the disciplines of neonatology, obstetrics and gynecology, respiratory medicine, geriatric medicine, hygiene, and public health reviewed the evidence on RSV prevention, and present here their considerations on RSV prevention in Italy. Our plea joins recent appeals from major Italian scientific societies, including recommendations from societies representing Obstetrics, Gynecology, Pediatrics and Neonatology to support the use of maternal vaccination to prevent LRTIs due to RSV [22]; the Board of the Italian “calendario della Vita”, which has expressed support for the use of long half-life mAbs for the prevention of RSV-associated LRTIs in newborns [23]; and the Italian Society of Hygiene, Preventive Medicine and Public Health (SItI), which supports the inclusion of RSV vaccines for adults aged 75 years or above in the national immunization calendar, together with the extension of vaccination coverage to include adults older than 60 years of age with chronic disease [24]. The Italian Society of Infectious and Tropical Diseases (SIMIT) [25], the Italian Ministry of Health [26], and the Italian Federation of Italian Pediatricians [27], all consider the implementation of passive and active preventive interventions against RSV-associated diseases to be urgent.

### RSV epidemiology

In temperate zones, RSV circulation is seasonal from late fall to early spring, peaking in winter [28, 29]. Before the SARS-CoV-2 pandemic, the RSV season in Italy usually extended from November to March, with a peak between January and February [30–32]; season to season alternation in the prevalence of RSV A and RSV B [33] is observed, as described in other temperate geographic areas [34].

**Fig. 1:**
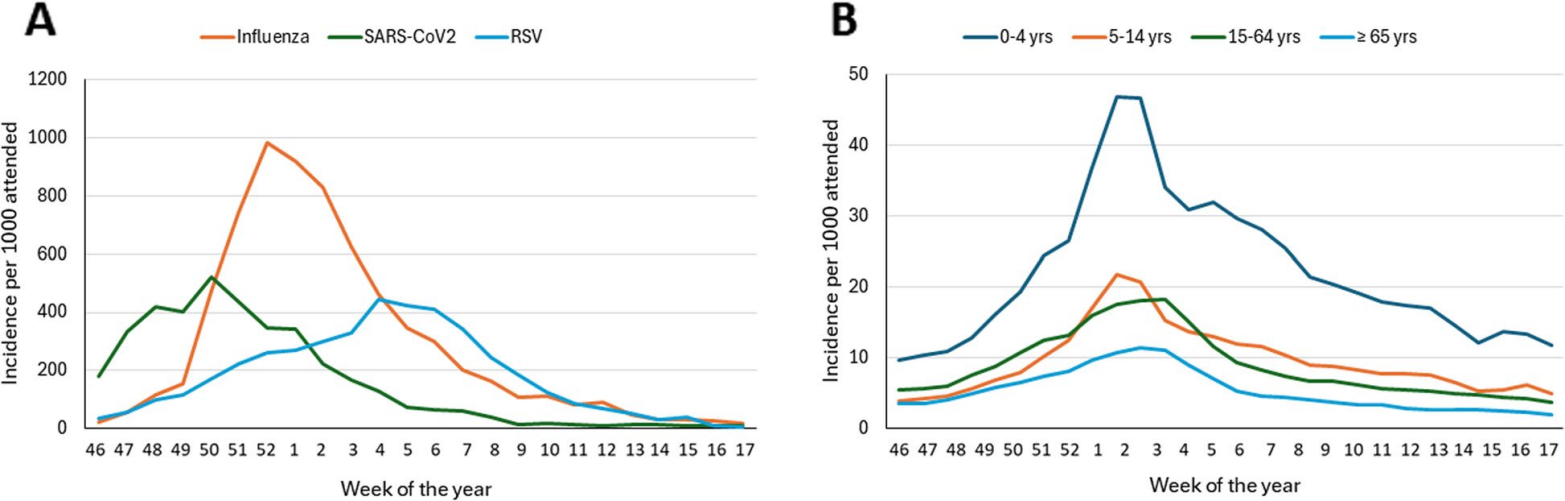
**a**, **b** Epidemiology of respiratory infections in Italy during the 2023–2024 season (Source Rapporto Virologico RespiVirNet May 2, 2024) [36]

Integrated epidemiological and virological surveillance is conducted in Italy by RespiVirNet (formerly InfluNet), coordinated by the Istituto Superiore di Sanità (ISS) with the support of the Ministry of Health, through a community-based network of sentinel clinicians and regional reference laboratories that use an ‘influenza-like illness’ (ILI) case definition [35]. In 2022, RSV was added to the list of respiratory viruses monitored by RespiVirNet (Fig. 1A). Data from the 2023–2024 season show that most RSV infections occurred in infants less than 4 years of age, but were also present in other age groups (Fig. 1B), as previously reported [31].

#### Considerations

The surveillance findings from the RespiVirNet sentinel network in Italy confirmed seasonal trends from hospital records in several Italian regions [37–40]. Enhanced surveillance will be critical for monitoring the circulating strains and predicting the start and end of RSV seasons, which can extend 2–3 weeks longer than the influenza season, and for measuring the impact of immunization on viral circulation and disease burden [41, 42].

The use of ILI case definition may under ascertain the prevalence of RSV infection, i.e., compared with the ‘acute respiratory illness’ (ARI) case definition, which better captures the incidence of bronchiolitis and pneumonia [43]. Moreover, clinical studies on nirsevimab and RSV vaccines have had prevention of LRTI/LRT disease (LRTD) as their endpoints; healthcare providers should therefore focus on pneumonia/LRTI, rather than ILI, presentations that are also better correlated with clinically relevant disease.

Increased respiratory virus testing, especially in symptomatic high-risk patients (e.g., in pediatric wards, elderly care facilities) may improve patient management, reduce transmission, and avoid unnecessary antibiotic use [44], while improving epidemiological data collection. A recent study on RSV circulation patterns revealed that air travel predicts the global spread of RSV [45].

## Burden of disease

### Infants and children

The Respiratory Syncytial Virus Consortium in Europe (RESCEU) estimates that annual RSV-associated hospital admissions in the European Union include approximately 250,000 children aged less than 5 years [46], including approximately 2% of otherwise healthy infants born at term [47].

Estimates for Italy from the RESCEU project suggest approximately 25,000 RSV-associated hospitalizations per year in children less than 5 years of age, corresponding to around 40% of all respiratory hospitalizations in that age group [46]. Retrospective analysis of administrative healthcare databases in Italy revealed that approximately 85% of RSV-related hospitalizations among children aged 0–5 years occurred in otherwise healthy children born at term, and that the annual incidence rate is 175–195/100,000 in this population [48].

There is also a substantial burden in the pediatric outpatient setting. A systematic review of six studies on the burden of RSV in Italian pediatric outpatients aged 0–60 months revealed that RSV positivity rates range from 18–41% of respiratory infections [49]. Retrospective analysis of a pediatric primary care database identified 7,956 episodes of bronchiolitis and 37,827 episodes of LRTI between 2012 and 2019 among children aged 0–24 months [50]. Only 2.3% of infections were coded as RSV-related, although outpatient viral testing is not common [51].

### Adults

The true burden of RSV infection in adults may be underestimated due to a low level of awareness among healthcare providers (HCPs) [52], limitations in case definitions [43, 53], and in diagnostic testing [54, 55]. Most adults have partial immunity to RSV, and reinfections may have a shorter duration and produce fewer viral particles in the nose. Consequently, tests based on nasal swabs can have a low detection rate [54, 56].

In Italy, 93.2% of RSV-associated hospitalizations in adults are recorded in patients over 65 years of age [46]. Infection with RSV causes substantial burden in otherwise healthy older adults, both community-dwelling and in residential care [6, 57–59], and in those with comorbidities [60]. In high-income countries, an estimated 470,000 RSV-associated hospitalizations occur per year among patients aged 60 years and above [61]; this number is estimated to be over 150,000 in the European Union [46]. Compared with younger adults, RSV infections in adults aged 60 years old and above result in a 3–5-fold increase in hospitalization rates and a 2-fold increase in emergency room and outpatient visits, with an increased risk of severe comorbidities and exacerbation of chronic degenerative conditions [61]. A systematic review of publications up to 2022 suggests that hospitalization and mortality rates due to RSV and influenza are similar in older adults [62]. Results from a US cohort study conducted before the availability of RSV vaccines suggest that RSV infections are associated with similar risk of hospitalization and death compared with influenza or COVID-19 in unvaccinated patients, but that RSV is associated with higher risk of hospitalization and death when compared to patients who had been vaccinated against influenza or COVID-19 viruses [63–65]. In up to 23% of cases, RSV infection is complicated by bacterial or other viral co-infections, further increasing the risk of hospitalization and death [66].

In Italy, systematic review and random effects meta-analysis of 35 observational studies conducted on adults tested for RSV infection identified an RSV-positivity prevalence of 4.5% in adults of any age, and 11.5% among those with immunosuppressive disorders [67]. Each year, an estimated 26,000 RSV-associated hospitalizations occur among Italian patients aged 60 years of age and above, resulting in an estimated 1,800 deaths [61].

#### Considerations

In addition to morbidity and mortality, RSV infections in infants and older adults are associated with a substantial economic burden [68–72]. In Italy, a retrospective study that assessed costs resulting from RSV-associated hospitalization in 310 otherwise healthy infants aged between 1 and 12 months admitted to the Bambino Gesù Children’s Hospital in Rome for bronchiolitis revealed total costs of €1,783,563 [73]. The mean cost was €5,753 (± 2,042) per RSV-associated hospitalization, compared with a mean cost of €5,395 (± 2,041) among 217 RSV-negative cases. Recently, a retrospective administrative database analysis conducted between 2010 and 2018 on 1,378 pediatric patients aged 0 to 5 years discharged after RSV-related hospitalization revealed that RSV infection was associated with higher annual direct healthcare costs per patient compared with the age-matched general population (€3,605 vs. €344) [48].

Although most costs related to RSV disease are direct, RSV disease can place a significant burden on families, including missed days of work for parents who need to care for sick children, transportation, and out of pocket medical expenses, as well as emotional stress [74–76]; it can also negatively impact quality of life (QoL) across the lifespan, including effects from asthma, possible neurological complications due to RSV-associated respiratory impairment [77–79], and exposure to drugs such as corticosteroids, bronchodilators and antibiotics, that are often erroneously prescribed off-label and may lead to avoidable costs, side effects and antibiotic resistance [80].

## Prevention strategies

The lack of effective treatment for RSV-associated LRTI underscores the need for preventive measures to minimize the clinical and economic burden of RSV while reducing its impact on health-related QoL and preserving healthcare system capacity during the peak respiratory virus season [80]. Beyond hygiene measures [81, 82] and promoting breastfeeding to protect infants against bronchiolitis during the first year of life [83], pharmacological strategies for protecting infants include passive immunization through maternal vaccination or administration of neutralizing mAbs to infants to delay an initial RSV infection or lessen its severity.

In temperate regions where RSV circulation is seasonal, passive prevention strategies may be administered either year-round, only during the RSV season, or during the season but via a ‘catch-up’ strategy at the start of the season to immunize infants born outside of the season. Meanwhile, pediatric vaccines are under development to provide coverage when protection from maternal antibodies or neutralizing mAbs wanes. Adults can benefit from active immunization through vaccination.

An improved understanding of RSV surface protein structures, neutralization-sensitive epitopes, and vaccine immunology has led to the development of safe and more effective agents for prevention. Respiratory syncytial virus surface glycoproteins G and F mediate host cell attachment and fusion, respectively. The major antigenic subtypes, RSV A and RSV B, are based on variations in the G protein sequence [84], and have similar clinical profiles [85]. Highly conserved site ϴ and V epitopes on the prefusion conformation of the fusion (F) protein are the primary binding sites of neutralizing antibodies in serum and targets for effective vaccines and mAbs [86]. Meanwhile, modifications in the Fc region of mAbs have extended serum half-life, allowing a single dose to protect infants for an entire RSV season.

Recent approval of three RSV prefusion F antigen vaccines [87–90], and the long-acting mAb nirsevimab [91–93], are welcome developments [17, 19–21] (Table 1).

As of this writing, the process leading to implementation of these prevention strategies had begun in several European countries; national recommendations are summarized in (Table 2).

**Table 2:** Snapshot of European countries where national recommendations for pharmacological preventive measures have emerged as of September 2024

Jurisdiction	Neonates and infants		Adults
	Abrysvo™	Beyfortus™	Abrysvo™/Arexvy™
*Europe (EMA)*	*Active immunization of pregnant women between 24–36 weeks of gestation*^a^	*Passive immunization with mAb – newborns and infants during their first RSV season*	*Active immunization of adults age* ≥ *60*^a^
Austria [94]	Administer from week 24–36, preferably September–March Not reimbursed	One-time application in year one of life September–March Not reimbursed	≥ 60 years or ≥ 18 years in adults at high risk Not reimbursed
Belgium Expected for 2024–2025 season	Administer from week 28–36, as a preferential window, for EDD between early September and end of March [95] Not reimbursed	All infants born: • from unvaccinated mothers • < 2 weeks after vaccination • prematurely, before week 30 [95] Synagis is reimbursed under certain restrictive conditions	≥ 60 years with at least one risk factor for severe RSV disease [96] Not reimbursed
France	Administer from week 32–36 [97] Reimbursed	Administered preferably before leaving the maternity ward [98] Reimbursed	≥ 65 years old with chronic respiratory pathologies (esp. COPD) or cardiac pathologies (esp. heart failure) likely to decompensate during an RSV infection, and ≥ 75 years with or without comorbidities [99] Not reimbursed
Germany		Infants born between April and September should receive nirsevimab in the fall, before their first RSV season Infants born during the RSV season must receive nirsevimab shortly after birth and before discharge from the birth center [100]	
Ireland [101]	Administer year-round or seasonal from week 24–36 Not reimbursed	Soon after birth during the RSV season; catch-up August–October Reimbursed	≥ 65 years before the onset of the autumn/winter RSV season
Luxembourg [102]	Administer from week 32–36 between September and February Reimbursed	Seasonal with catch-up for infants < 6 months Reimbursed	–
Netherlands	–	Universal seasonal with catch-up [103]	–
Norway [104]		–	Can be considered for age ≥ 60 years with an underlying disease
Poland [105]	–	–	≥ 60, if prescribed
Slovenia [106]	Administer from week 24–36. Reimbursed		
Spain	–	All infants < 6 months born April 1, 2024–March 31, 2025 [107] Reimbursed	–
Sweden [108]	Administer from week 30–36	–	≥ 75 years with comorbidities and ≥ 60 years with certain underlying diseases
Switzerland	–	Authorized 22 December 2023 • before or during first RSV season • in infants age ≤ 24 months at risk of severe RSV disease in their second RSV season [109]	Authorized 2 May 2024 [110]
United Kingdom [111]	Administer year-round from week 28–36 Full reimbursement starting Sept 1, 2024	JCVI: maternal immunization and mAb strategies are both suitable for universal RSV program Not reimbursed	JCVI advises a program for adults aged ≥ 75 years Reimbursement in place for RSVpreF

– recommendation not available

*COPD* chronic obstructive pulmonary disease, *EDD* expected due date, *EMA* European Medicines Agency, *JCVI* Joint Committee on Vaccination and Immunisation, *LRTD* lower respiratory tract disease, *mAb* monoclonal antibody, *RSV* respiratory syncytial virus

^a^Can be administered concomitant with seasonal influenza vaccine. A minimum interval of two weeks is recommended between administration of Abrysvo™ and administration of a tetanus, diphtheria and acellular pertussis vaccine (Tdap)

### Protecting newborns and infants

#### RSVPreF—bivalent prefusion F vaccine (RSV Subgroups A + B antigens), maternal indication

The recombinant bivalent prefusion F (RSVPreF), nonadjuvanted vaccine Abrysvo™ (Pfizer) is indicated for maternal vaccination administered as a single dose to pregnant women in the late second or the third trimester (gestational week 24–36), with a European Medicines Agency (EMA)-recommended minimum interval of two weeks between vaccine delivery and administration of the tetanus, diphtheria and acellular pertussis vaccine (Tdap) [17]. RSVPreF provides passive immunity through transplacental transfer of antibodies that protect the infant from LRTIs through the first 6 months of life [17]. In the phase 3 MATISSE study, 7,358 pregnant women were randomized (1:1) to receive RSVPreF vaccine or placebo at gestational week 24 to 36, meeting the pre-specified primary endpoint of medically attended severe RSV-associated LRTI, with a vaccine efficacy of 81.8% at 90 days and 69.4% at 180 days [89].

RSVPreF was well tolerated, with injection site pain and muscle pain being the most frequent adverse events (AEs) in the vaccine group. Adverse events in infants were similar in the vaccine and placebo groups. There was a non-significant imbalance in preterm deliveries, compared with placebo (201 [6%] vs 169 [5%]) [17]; however, further analysis failed to show a correlation between vaccination timing and premature birth, but revealed that the imbalance may be driven by results from a small subgroup of upper-middle income countries. Although not statistically significant, the signal drew attention because a similar observation had halted enrollment in a clinical study and resulted in discontinuation of the GSK candidate maternal vaccine (RSVPreF3-Mat; NCT04605159) [112]. As a precautionary measure, the US Food and Drug Administration recommended administering RSVPreF between gestational weeks 32–36 [113], while the EMA recommends the vaccination timing used in the MATISSE study (24–36 weeks gestation) [17]. Further analysis should address the relative risks and benefits of vaccine delivery, considering that severe RSV infection during pregnancy is associated with a substantial increase in the risk of preterm delivery (OR 3.6 [95% CI, 1.3–10.3]) [114], maternal pulmonary and respiratory complications at delivery (adjusted OR 1.82) [115], and potential immunoinflammatory responses in the fetus [116].

Real-world evidence from a cohort of 2973 pregnant women, of whom 1026 (35%) received prenatal RSVPreF vaccination between weeks 32 and 36 of gestation (mean gestational age at vaccination 34.5 weeks), did not reveal an increase in the risk of preterm birth among women who received the RSVPreF vaccine. Sixty of the pregnant women who received the RSVPreF vaccine during the 2023–2024 RSV season in two New York City hospitals had preterm births (5.9%), compared with 131 of the unvaccinated women (6.7%) [117].

Preliminary post-introduction safety findings presented by the US Centers for Disease Control suggest that the incidence of preterm births among women who received the RSVPreF vaccine between gestational weeks 32 and 36 during the 2023–2024 RSV season was 4.1%, which is within the expected range of preterm births at this gestational age (3.1–6.1%); other reported AEs occurred with frequencies similar to those observed in pre-licensure trials [118].

#### Long-acting RSV monoclonal antibody (nirsevimab)

The anti-RSV mAb nirsevimab (Beyfortus™) is approved for the prevention of RSV-associated LRTIs from birth through the first RSV season [19], while another anti-RSV mAb is in clinical development (MK-1654, clesrovimab) [119, 120]. Nirsevimab provided protection for up 5 to 6 months after a single dose. A recent study demonstrated a longer durability of neutralizing RSV antibodies after a single dose of nirsevimab, with values more than 7-fold higher than baseline levels of naturally acquired maternally transferred neutralizing RSV antibodies at day 361 [121].

The pivotal MELODY study assessed the safety and efficacy of nirsevimab compared with placebo in term and late preterm infants (gestational age 35 weeks or over). Efficacy was 74.5% for the primary endpoint of RSV-confirmed medically assisted LRTIs 150 days after injection, and 62.1% for the secondary endpoint of hospitalizations for RSV-related LRTIs 150 days after injection; no efficacy data were presented at intermediate timepoints, and no safety issues were reported [122]. The use of nirsevimab is supported by results of the MEDLEY trial, which confirmed its safety compared with palivizumab in infants at high risk for severe RSV disease [93]. Universal immunization of newborns with nirsevimab has been implemented in France, Luxembourg and Spain, along with the autonomous Valle d’Aosta Region in Italy and in the autonomous Madeira region in Portugal.

Early estimates from surveillance in 3 regions of Spain where immunization coverage was 79 to 99% showed that nirsevimab was effective in preventing hospitalizations for RSV-associated LRTI in infants [123]. Effectiveness was 70.2% when hospitalizations among immunized and unimmunized infants were compared (test-negative design), and 84.4% when vaccinated and unvaccinated individuals were monitored over time (screening method).

Surveillance data from the Galicia region of Spain revealed coverage of 84.8% among infants in the catch-up cohort born out of season (1 April to 24 September 2023), and 92.4% among infants born in season, corresponding to a dramatic reduction in RSV hospitalizations compared with previous seasons [124]. In the US, population-based surveillance of ARI in 4 centers identified 699 infants hospitalized with ARI between October 1, 2023 and February 29, 2024 [125]. Using a test-negative assessment, nirsevimab was 90% effective against RSV-associated hospitalization. Additional supporting real-world evidence comes from studies in Luxembourg [126], Spain [127], France [128], and the autonomous Valle d’Aosta Region in Italy [129]. An early summary of the real-world effectiveness of nirsevimab suggested a pooled estimate of 88.4% against the occurrence of hospital admission due to RSV, with a 2-fold increase in the risk of breakthrough infections in studies with observation times of 150 days or more compared with studies lasting less than 150 days [130].

#### Considerations on implementation

##### Seasonality and timing

**Fig. 2:**
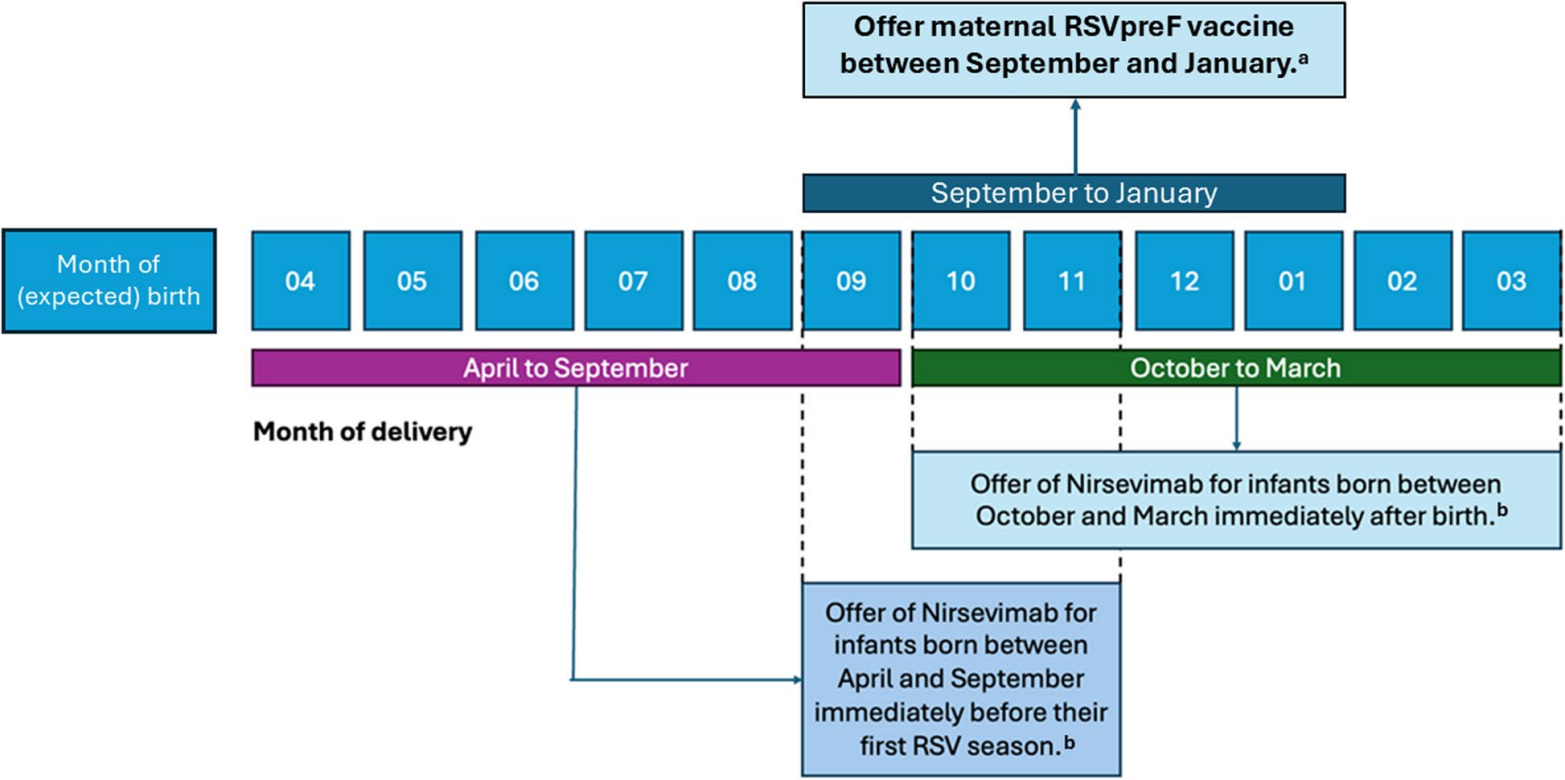
Summary of recommendations for passive immunization to protect newborns from RSV disease. (Modified from the Standing Committee on Vaccination at the Robert Koch Institute (STIKO) 2024 Guidelines [100], based on US Centers for Disease Control’s Advisory Committee on Immunization Practices (^a^ACIP) 2023 [133] and ^b^STIKO 2024 [100]). RSV, respiratory syncytial virus

Infants born just before or during the RSV season have a much higher risk of RSV infection in their first year, compared with those born after the season ends [131]. This observation suggests that a seasonal prevention strategy may be more cost-effective than year-round coverage [132], as recently recommended in guidelines from the Standing Committee on Vaccination at the Robert Koch Institute (STIKO) [100] (Fig. 2).

However, a year-round vaccination program may be simpler to implement, which may facilitate uptake. The relatively predictable seasonal trend and the surveillance system used in Italy are amenable to a seasonal approach, although the recent disruption of RSV seasonal trends associated with the SARS-CoV-2 pandemic should be monitored carefully.

Success will also depend on the duration of protection. Maternal vaccination with RSVPreF in the late second or the third trimester provided protection for up to 6 months [17], as determined by the primary efficacy endpoint met in the pivotal MATISSE study: medically attended severe RSV-associated LRTI in infants within 180 days after birth [89]. Vaccine efficacy 6 months after birth was 69.4% against medically attended severe LRTIs [89].

The duration of protection after maternal vaccination should be evaluated more fully by new research, including the contribution of maternal antibodies transferred in breast milk. A systematic review of 19 studies showed that infants who were not breastfed had an increased risk of severe RSV-associated acute lower respiratory infection and hospitalization, whereas those who were breastfed exclusively for more than 4 to 6 months had significantly reduced hospital admissions, length of stay, supplemental oxygen demand, and admissions to intensive care [134]. A correlation was found between RSV-associated acute respiratory infections in infants and levels of anti-RSV pre-F IgG, but not IgA antibodies in breast milk, suggesting a role for pre-F IgG in protection after maternal RSV vaccination [135].

##### Organizational aspects

Both maternal vaccination and passive immunization with mAbs are single-dose solutions that provide effective protection against RSV during the most critical first six months of life. In most cases, either maternal vaccination with RSVPreF or immunoprophylaxis with nirsevimab will be used; however, administration of nirsevimab to infants born after maternal vaccination may be required if they are born less than 14 days after vaccination, have hemodynamically significant congenital heart disease, or undergo cardiopulmonary bypass/extracorporeal membrane oxygenation [133].

The choice between passive immunization strategies should consider the estimated due date with respect to the projected start of the RSV season. For delivery dates predicted after the season (April–September), it may be appropriate to forego maternal vaccination and administer nirsevimab in October; however, this approach is not currently available in Italy and there is some risk that infants may not receive protection before the subsequent RSV season. Attention should also be paid to infant body weight – two doses may be needed for larger infants.

Ongoing universal immunization campaigns in France and Spain have adopted a seasonal approach to administering nirsevimab before discharge from the maternity ward to all infants born during the season, starting in fall 2023, whereas those born out of season who are less than 6 months old at the start of the next season will receive nirsevimab in the subsequent fall [98, 136].

Regarding subsequent pregnancies, currently, there is no evidence to inform whether a woman who was vaccinated during a previous pregnancy should be revaccinated during subsequent pregnancies; therefore, until evidence is available, such infants should receive passive immunization with nirsevimab as soon as possible after birth.

##### Coadministration – effect on vaccine responses.

Practical considerations for effective vaccine implementation include keeping the number of vaccination calendar visits unchanged by administering multiple vaccines at each visit. Simultaneous administration of RSVPreF with Tdap or influenza vaccines has been shown to slightly decrease the immune response to the pertussis components of Tdap (noninferiority criteria not met) in healthy, nonpregnant women 18‒49 years of age [137]. The clinical relevance of this minor reduction in vaccine response is not known, but warrants monitoring [138]. The EMA recommends that RSVPreF can be administered with the seasonal influenza vaccine, but has recommended an interval of at least 2 weeks between administration of RSVPreF and the Tdap vaccine [17], whereas the US Centers for Disease Control’s Advisory Committee on Immunization Practices (ACIP) allows simultaneous administration with other recommended vaccines, including Tdap and influenza, without regard to timing [133], in accordance with their General Best Practices Guidelines for Immunization [139].

### Protecting older adults

Currently, three prefusion F vaccines (Table 3) are approved for the prevention of LRTI after single dose vaccination of adults aged 60 years of age or above, including two recombinant vaccines (RSVPreF [17] and RSVPreF3 [20]), and a messenger RNA-based vaccine (mRNA-1345 [21]). The success of their implementation will require careful consideration and guidance from scientific societies concerned with this population. Nonetheless, a preliminary analysis of available randomized controlled trials has suggested that all commercially available vaccines are comparable in terms of efficacy [140], at least in the first RSV season, while some heterogeneities have been identified in follow-up seasons.

**Table 3:** Summary of characteristics of approved RSV vaccines for older adults

	**RSVPreF** [17]	**RSVPreF3** [20]	**mRNA-1345** [21]
**Technology**	Protein	Protein	mRNA
**Targeted antigen**	Prefusion F protein from RSV subgroups A + B (bivalent)	Prefusion F protein from RSV subgroup A	Prefusion F protein from RSV subgroup A
**Adjuvated**	No	Yes	No
**Content**	60 µg + 60 µg prefusion F protein	120 µg prefusion F protein	50 µg mRNA for prefusion F protein
**Number of doses**	Single	Single	Single

A real-world study conducted in the US used a test-negative design to assess electronic health records from 28,271 hospitalizations and 36,521 emergency department accesses, demonstrating the effectiveness of vaccination against RSV-associated hospitalization and emergency department access by non-immunocompromised adults aged at least 60 years who presented with RSV-like illness during the 2023–2024 RSV season, revealing vaccine effectiveness of 80% against RSV-associated hospitalization and 77% against emergency department access, reporting similar effectiveness estimates for RSVPreF and RSVPreF3 [141]; mRNA-1345 was licensed by the US FDA in May 2024, after the study had ended.

#### RSVPreF—bivalent prefusion F vaccine (RSV Subgroups A + B antigens), older adult indication

The Pfizer recombinant, nonadjuvanted bivalent prefusion F vaccine indicated for adults aged at least 60 years has the same formulation as the maternal vaccine [17]. In the pivotal RENOIR study, 36,862 adults aged 60 years and above were randomized (1:1) to RSVPreF or placebo, resulting in a vaccine efficacy against LRTI with at least 3 symptoms of 88.9% in the first season [87]. Efficacy was 77.8% in the second season, with similar results for RSV A and RSV B [142]. The EMA indicates that RSVPreF can be administered concomitantly with the seasonal influenza vaccine (QIV, surface antigen, inactivated, adjuvanted) [17]. RSVPreF was well tolerated, with the most frequently reported adverse reaction being vaccination site pain (11%) [17].

#### RSVPreF3—prefusion F vaccine (RSV Subgroup A antigen) with adjuvant AS01E

The GSK recombinant prefusion F vaccine with adjuvant AS01E is indicated for adults aged 60 years and above [20]. In the pivotal AReSVi-006 trial, 24,966 participants were randomized (1:1) to RSVPreF3 or placebo at the start of the RSV season; the primary endpoint was prevention of RSV-related LRTD during one RSV season [88]. Vaccine efficacy was 82.6% after one season (median follow-up 6.7 months), and was similar for RSV A and B subtypes (84.6% and 80.9%, respectively). Efficacy against RSV-related LRTD was 56.1% in the second season (76.4% against RSV A and 43.9% against RSV B) [143]. The most frequently reported adverse reactions with RSVPreF3 were injection site pain (61%), fatigue (34%), myalgia (29%), headache (28%), and arthralgia (18%), which were usually mild/moderate in intensity and resolved within a few days [20].

#### mRNA-1345—prefusion F lipid nanoparticle-encapsulated mRNA vaccine (RSV Subgroup A antigen)

The Moderna mRNA vaccine encodes a stabilized prefusion F (RSV A) and is indicated for adults over 60 years of age [21]. The pivotal phase III ConquerRSV study was conducted in 35,541 adults aged 60 years or above randomized (1:1) to mRNA-1345 or placebo, with two co-primary efficacy endpoints of RSV-LRTD with at least 2 signs or symptoms, and RSV-LRTDs with at least 3 signs or symptoms. At interim analysis (median follow-up 112 days) efficacy against RSV-LRTD with at least 2 signs or symptoms was 83.7% (91.7% for RSV A, and 68.5% for RSV B); efficacy against RSV-LRTDs with at least 3 signs or symptoms was 82.4% (90.0% for RSV A, and 71.5% for RSV B) [90]. Local adverse reactions were more common in the mRNA-1345 group, compared with the placebo group (58.7% vs. 16.2%), as were systemic adverse reactions (47.7% vs. 32.9%); however, most reactions were mild to moderate in severity [90].

### Considerations on implementation

#### Vaccine timing

Because the duration of protection is not yet known, vaccines should be administered before the start of the RSV season. While preliminary results showing protection in a second RSV season are encouraging [142, 143], continuing surveillance is needed to determine the duration of effective protection and whether additional doses are needed. Protection lasting more than 2 years would preclude the need to synchronize vaccination with seasons and would improve cost-effectiveness.

#### Coadministration – effect on vaccine responses

Concomitant administration of RSV and seasonal influenza vaccines is an acceptable practice that does not affect immunogenicity and could reduce health-care visits and increase vaccination uptake. Coadministration of RSVPreF3 with a quadrivalent inactivated influenza vaccine met criteria for non-inferiority of the immune responses between the influenza and coadministration groups; however, responses were numerically lower compared with separate administration groups [144]. Coadministration of RSVPreF3 with the Tdap vaccine resulted in the expected response against RSV, but a somewhat blunted response to the pertussis component of Tdap [145]; similar observations were reported with RSVPreF when combined with Tdap (*see also* Protecting newborns and infants, above**)** [137], and when adjuvanted or non-adjuvanted versions of the RSVPreF vaccine were combined with a seasonal inactivated influenza vaccine [146]. Coadministration of the RSV mRNA-1345 vaccine with a quadrivalent inactivated influenza vaccine or SARS-CoV-2 mRNA-1273.214 resulted in robust immunogenicity for all antigens [147].

#### Promoting awareness and acceptance

Reducing the burden of RSV is a public health priority. Preventive measures are not useful if they are not accepted. Education and advocacy are key to promoting awareness and acceptance. Only 43% of US adults surveyed were aware of RSV [148], while studies on knowledge, attitudes and practices among Italian medical professionals have hinted at low awareness of the potential severity of RSV infections in certain high-risk groups, including older adults [149]. Knowledge gaps, particularly among healthcare workers, can ultimately result in vaccine hesitancy from insufficient information or misinformation that in turn hinders vaccine uptake. This phenomenon has led to the re-emergence of vaccine-preventable diseases, such as the recent measles outbreak in Italy [150]. In fact, the Strategic Advisory Group of Experts on Immunization (SAGE) of the World Health Organization found that increasing knowledge and awareness of vaccines through educational initiatives, especially in the context of a hospital procedure or existing medical process, can be helpful [151]. Vaccine mandates with associated sanctions, the use of reminders, and interventions that improve access and convenience also tended to meet with success; however, the least successful interventions were those using posters, websites, media releases, or radio announcements, as well as those based on financial incentives [151]. A simplified model summarizes the factors contributing to hesitancy as ‘complacency, confidence, and convenience’ – *complacency* due to lack of awareness of the potential risks from RSV infections, low *confidence* that the vaccine is safe, and (in)*convenience* associated with medical visits, costs, etc. [152].

A survey of expecting and recent first-time parents in Europe, the US, and China revealed that HCP recommendations and inclusion in an immunization program will be crucial for the acceptance of infant immunization with mAbs [153]. Official recommendations that explain the importance and safety of vaccination in pregnancy are crucial for promoting vaccine acceptance among pregnant women [154].

Institutional barriers can also result in low vaccine uptake [155]. Immunization programs within existing care services may improve vaccine coverage; for convenience, existing maternal immunization visits or routine third trimester screenings in the vaccination window can be used for maternal vaccines, while mAbs can be administered in the maternity ward or during early visits for newborns. Influenza or pneumococcal vaccine programs can be leveraged for vaccinating older adults against RSV [156].

Access to vaccinations can be made more convenient for older adults by offering them in clinics, pharmacies, workplaces, and other accessible locations at convenient times [157], as well as by offering co-administration of two or more vaccines during the same appointment [158]. Building and maintaining confidence requires ongoing surveillance data demonstrating safety, and evidence to support continuing effectiveness.

## Conclusions

Effective new immunizing agents have the potential to make severe RSV disease an immunization-preventable disease. Given the associated disease burden, it is essential to move quickly to deploy these agents in vulnerable populations, considering epidemiological trends, healthcare infrastructure, and planning for long-term sustainability. In Table 4, we present our considerations for implementing preventive measures in Italy.

**Table 4:** Administration strategies for protecting vulnerable populations in Italy

**Protecting infants** The efficacy and safety of the mAb nirsevimab and the maternal vaccine RSVPreF are similar. Generally, infants should be passively immunized with one of these strategies, but in rare circumstances certain infants may benefit from a dual approach.
**Maternal RSV vaccination**
All pregnant women are eligible for vaccination, except immunocompromised individuals; in women at high risk of preterm delivery, delay administration until after 32 weeks of gestation as a precautionary measure while awaiting clarification regarding the potential risk of premature birth. The need for revaccination with subsequent pregnancies has not been established.
Administer a maternal RSV vaccine between gestational weeks 24 and 36, according to local recommendations. Follow a year-round administration strategy given that RSV circulation at the time of delivery may not be predictable; seasonal administration may miss opportunities for protection, whereas a year-round strategy – provided it’s feasible and cost-effective – may maximize the population benefits.
• Infants born < 2 weeks after maternal vaccination should be considered at risk of limited protection and hence eligible for nirsevimab. Infants at high risk for severe RSV disease^a^ born out of season to vaccinated mothers should also receive nirsevimab before the start of their next RSV season.
**Nirsevimab administration**
• Infants born at the start or during the RSV season who have not been passively immunized through maternal vaccination should receive nirsevimab before leaving the maternity ward. • Infants born after the RSV season who have not been passively immunized through maternal vaccination should receive nirsevimab at the beginning of the next RSV season. • Infants at high risk for severe RSV disease^a^ should also receive nirsevimab before the start of their second RSV season.
**Protecting adults age ≥ 60 years and adults of any age with high-risk comorbidities**^**b**^ The RSVPreF and RSVPreF3 vaccines have similar efficacy and safety, and both provide some protection for at least two seasons.
• Eligible adults should receive one of these vaccines at any convenient time in the routine immunization schedule; whenever possible, schedule this vaccination before the start of RSV season. • Evidence shows protection for at least two seasons. Ongoing studies will determine the duration of effective protection, potential usefulness of revaccination and optimal vaccination interval.

*mAb* monoclonal antibody, *RSV* respiratory syncytial virus

^a^Infants considered to be at high risk for RSV disease include those currently eligible for palivizumab [18]

^b^Adults of any age who are immunocompromised, have diabetes or a chronic respiratory-, cardiovascular-, hepatic-, hematologic-, or renal disease that increases their risk of severe RSV disease and/or adults in long-term care facilities

Enhancing respiratory virus surveillance using the most appropriate case definition and extending it beyond the peak season will be essential to ensuring that seasonal RSV trends are accurately detected/predicted, and that the impact of prevention strategies can be assessed. Enhanced surveillance, combined with widespread use of more sensitive testing and continuing research would allow better definition of the burden of RSV in the outpatient setting and may identify vulnerable populations and/or risk factors.

Providing access to preventive agents alone is not sufficient to ensure successful implementation. Access must be accompanied by coordinated and targeted educational initiatives and advocacy to promote acceptance of active and passive immunization strategies by HCPs and the target population. The Italian healthcare system is regional, but it will be important to promote initiatives with a national prevention plan and develop educational materials on RSV burden and the benefits of immunization in various settings.

## Data Availability

No datasets were generated or analysed during the current study.

## Abbreviations

ACIP: Advisory Committee on Immunization Practices (US CDC)
AE: Adverse event
ARI: Acute respiratory illness
COPD: Chronic obstructive pulmonary disease
EDD: Expected due date
EMA: European Medicines Agency
EU: European Union
HCP: Healthcare professionals
IgG1κ: Immunoglobulin G1 kappa
ILI: Influenza-like illness
ISS: Istituto Superiore di Sanità
JCVI: Joint Committee on Vaccination and Immunization
LRTD: Lower respiratory tract disease
LRTI: Lower respiratory tract infection
mAb: Monoclonal antibody
PCR: Polymerase chain reaction
QIV: Quadrivalent influenza vaccine
QoL: Quality of life
RESCEU: Respiratory Syncytial Virus Consortium in Europe
RSV: Respiratory syncytial virus
RSVPreF: Respiratory syncytial virus prefusion F
SAGE: Strategic Advisory Group of Experts on Immunization
SARS-CoV-2: Severe acute respiratory syndrome coronavirus 2
STIKO: Standing Committee on Vaccination (Robert Koch Institute)
Tdap: Tetanus, diphtheria and acellular pertussis vaccine
